# Molecular mechanisms of cardiovascular benefits of exercise: Running for cover from heart disease

**DOI:** 10.21542/gcsp.2016.3

**Published:** 2016-03-31

**Authors:** Mohamed Hassan, Yasmine Aguib, Magdi Yacoub

**Affiliations:** Aswan Heart Center, Aswan, Egypt

The benefits of exercise have been recognized since ancient times (Figure 1). Physically active men and women have an approximately 30% lower risk of death compared with inactive people. Several trials have recently shown the favorable impact of exercise on survival and quality of life. In the PARIS study,^1^ four months of endurance exercise training in elderly patients with heart failure and preserved ejection fraction caused a significant improvement in peak exercise capacity. Moreover in the Copenhagen City Heart Study,^2^ jogging up to 2.5 h per week at a slow or average pace and a frequency of 3 times per week was associated with a significant increase in survival (6.2 years in men and 5.6 years in women). These findings imply that exercise improves peripheral vascular, microvascular, and/or skeletal muscle functions and causes an increase in oxygen transport and utilization by the active skeletal muscle.^1^ However, the exact molecular mechanisms of the cardiovascular benefits of exercise remained largely unknown until very recently. Two recent reports serve to shed some light on the potential role for irisin and miRNA-222 in this subject.^3, 4^

## Irisin and exercise

Irisin – named after the Greek messenger goddess Iris – is a newly identified polypeptide myokine, secreted from skeletal muscle into the circulation in response to physical exercise.^5^ Excercise induces the production of a transcriptional co-activator –*peroxisome proliferator-activated receptor* γ *co-activator* 1α (PGC-1α) – in muscles which stimulates mitochondrial biogenesis, angiogenesis, fibre-type switching towards the more oxidative and high endurance type IIa and type I fibers, as well as the expression of a type 1 transmembrane protein called fibronectin type III domain containing 5 (FNDC5).^6^ In 2012, Bostrom et al. described irisin as a cleaved and secreted part of FNDC5, which is released into the circulation.^5^

After the debate regarding the existence of irisin in humans, and the claims that FDNC5 gene is a pseudogene,^7^ plasma irisin levels have been recently demonstrated to increase progressively in response to increasing exercise workloads.^3, 8^ When measured by mass spectroscopy in an unbiased and antibody-independent manner, irisin was detected in the blood at a level of 3.6 ng/ml in sedentary individuals which increases significantly to 4.3 ng/ml in individuals undergoing aerobic training.^3^

Irisin binds to white adipose tissue (WAT) and upregulates the expression of peroxisome proliferator-activated receptor α (PPAR-α) and mitochondrial brown fat uncoupling protein1 (UCP1), which subsequently induces brown adipose tissue (BAT) gene program to transform WAT cells to beige adipocytes – known as ‘browning’ of subcutaneous WAT (Figure 2).^9, 10^ This causes a significant increase in total body energy expenditure and thermogenesis.

**Figure 1.**
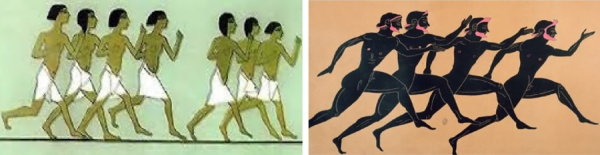
Ancient Egyptian and Greek runners.

**Figure 2.**
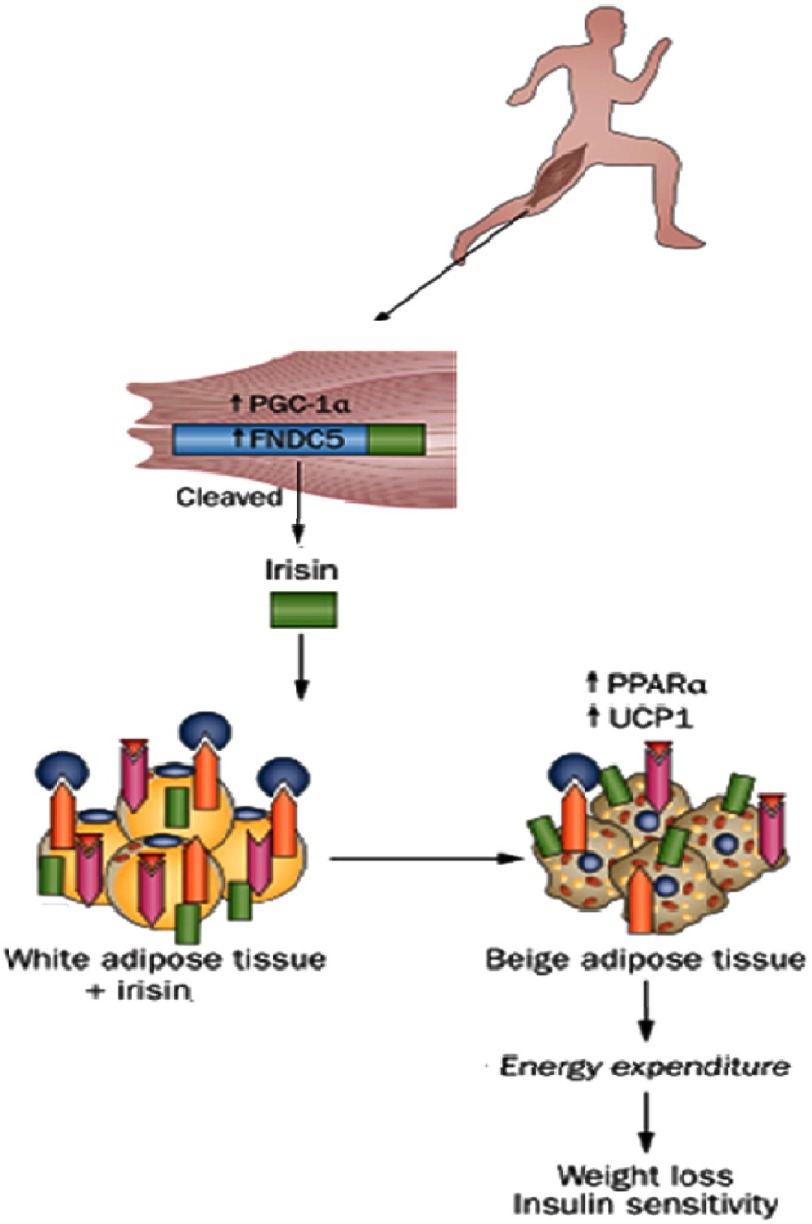
Crosstalk between skeletal muscles and adipose tissue: Exercise induced browning of white adipose tissue. (adapted from [9]). FNDC5, fibronectin type III domain-containing protein5; PGC-1α, peroxisome proliferator-activated receptor γ co-activator 1α; PPARα, peroxisome proliferator activated receptorα; UCP1, mitochondrial brown fat uncoupling protein1.

The crosstalk between skeletal muscles and adipose tissue mediates some of the beneficial effects of exercise on energy expenditure and metabolism, which improves glucose homeostasis, insulin sensitivity, and helps to reduce body weight.^9, 10^ This may highlight the therapeutic potential of irisin in fighting obesity and diabetes mellitus.

## miR-222 and exercise

MicroRNAs (miRNAs, miRs) are short, non-coding 18-25 nucleotide long RNAs which bind and inhibit mRNA, thereby regulating gene expression post-transcriptionally. They generally bind to the 3’-UTR (untranslated region) of their target mRNAs and repress protein production by destabilizing the mRNA which leads to translational silencing.^11^ The first report of a miRNA, lin-4, that regulates the development of *Caenorhabditis elegans* dates back 20 years.^12^ Our knowledge that microRNA targeting of mRNA can occur in a conserved (or an unconserved fashion) made it clear to us, that when we further study regulation of gene expression, it is very likely to find that most of the genes are at some point regulated by miRNAs.

## miRNA in cardiac development and physiology

In the cardiovascular system, miRNAs have been shown to be pivotal regulators of development and physiology and, thus, are directly involved in the pathophysiology of many cardiovascular diseases.^13–18^ Multiple miRNAs are implicated in different aspects of cardiovascular development. Animal studies show that miR-1 (miR-1-1, mir-1-2) targets, amongst other transcription factors (TFs), Hand2, a promoter of ventricular cardiomyocyte expansion, and thus negatively regulates cardiac growth during mouse development by inhibiting its translation.^19–21^ MiR-133 is another highly conserved miRNAs derived from a common precursor transcript with mir-1, and also exhibits cardiac- and skeletal- muscle specific expression during development and adult life.^20–23^ For example, studies in miR-133a-1/ miR-133a-2 double mutant mice showed that miR-133a gene targets include Cyclin D2 and Serum Response Factor and influences the regulation of cell cycle control and the activation of the smooth muscle gene program.^24^ miRNA profiling studies in human embryonic stem cell-derived cardiomyocytes (hESC-derived CMCs) led to the confirmation and identification of numerous miRNAs that promote cardiac specification. The roles of miR-1 and miR-133 as well as the novel miR-499 were further characterized. Overexpression of mir-1 in hESCs led to upregulation of the TF GATA4, which is crucial during early heart development, miR-499 overexpression in hESCs led to elevated protein levels of the cardiac TF MEF2C, which is required for cardiac contractile gene activation and for the structural development of the heart.^25–28^

### miRNA in cardiac pathophysiology

miRNA are also increasingly associated with cardiac pathology, they exert wide-ranging functions and regulate entire gene expression networks.^29^ A recent review by Kalozoumi et al. highlights the role of miRNA in heart failure pathology.^18^ In order to understand the miRNA signature in human failing hearts miRNome studies were conducted using advance microarray, next generations sequencing and other techniques.^30–34^

On the one hand miRNA expression profiles resulting from different cardiac stresses displayed overlaps suggesting common responses to different stimuli. On the other hand there are also miRNAs that show a unique expression profile related to a specific cardiac stress. For instance, miR-23a has been shown to promote and miR-1 to inhibit cardiac hypertrophy, miR-24 and 133a have been shown to negatively regulate fibrosis.^34^

**Figure 3.**
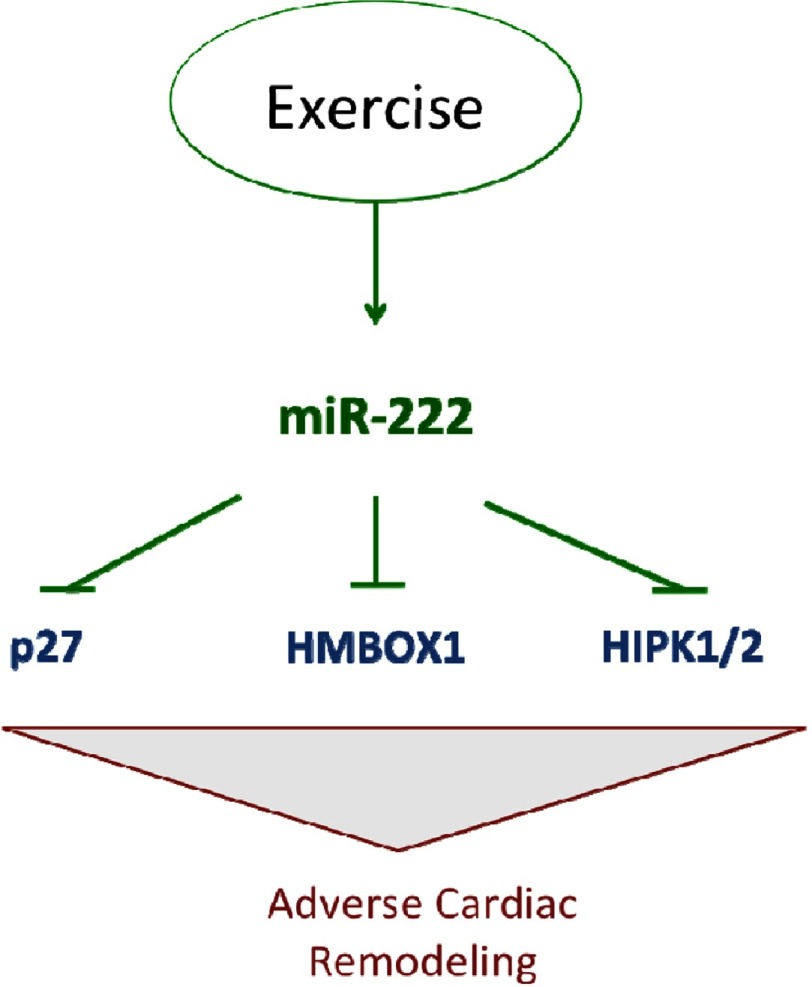
miR-222 and exercise. Adapted from reference [4].

These four miRNAs are also amongst the most abundant miRNAs in the HF samples that have been described in previous CVD studies. Interestingly, miR-145 emerged as a new player in left ventricle pathological remodeling and was also identified as one of the most abundant miRNAs in the heart. Its expression, both in DCM and HCM, was significantly increased. This miRNA is involved in smooth muscle cell fate and plasticity where it functions to regulate the quiescent versus proliferative phenotype of smooth muscle cells.^35^

In the heart, miR-145 is downregulated following acute myocardial infarction and seems to be involved in cardiac remodeling via the regulation of Dab2 expression.^36^ Upregulation of the previously mentioned miR-499 in human hypertrophied and failing hearts was associated with decreased expression of several predicted targets, such as AKT and MAPKs. In mice, miR-499 was even sufficient for the induction of HF and acceleration of the pathological remodeling, upon pressure overload.

### miR-222, exercise, and cardiac remodeling

Cardiac (or ventricular) remodeling is defined as alteration in the structure (dimensions, mass, shape) of the heart in response to hemodynamic load and/or cardiac injury and may be described as physiologic or pathologic,^37^ or as adaptive or maladaptive.^38^

The remodeling process frequently includes myocardial hypertrophy, which may occur with or without an increase in overall myocardial mass. In physiologic remodeling, physiologic stimuli, such as exercise, cause compensatory changes in the dimensions and function of the heart, which also have important effects on the cellular and molecular level.

The multiple roles of miRNAs, as described above for miR-1, miR-133 and miR-499, in different cardiac health and disease scenarios, namely hypertrophy and both in physiological and pathological scenarios, show us how important it is to exploit the beneficial effects of miRNA in cardiac remodeling, e.g. in the context of exercise. As the molecular mechanisms underlying the effect of exercise are quite unknown, it was very tempting for many researchers to elucidate the effect of exercise on miRNA levels and miRNA regulated gene expression.^39–42^ An upregulation of the miR-29 family by physical training was associated with a significant decrease in left ventricular collagen gene and protein levels and was also accompanied by improvement of ventricular compliance and cardiac function.^42, 43^ Also, downregulation of miRNA-1, -133a, -133b and -214 were observed in the exercised heart.

A recent study by Liu and colleagues has shed new light on mechanisms through which exercise promotes adaptive (healthy) heart growth and blunts pathologic cardiac remodeling, unraveling a critical role for the microRNA miR-222.^4^ The study provides strong evidence that exercise upregulates miR-222 which is important for cardiomyocyte and hence cardiac growth and mitigation of cardiac pathologies. Downstream targets modulating cardiomyocytes phenotypes were identified, including Cyclin-dependent kinase inhibitor 1B (p27), Homeodomain interacting protein kinase 1/2 (HIPK1) and Homeobox containing 1 (HMBOX1) (Figure 3). *In vivo* inhibition of miR-222 exercise blocked cardiomyocyte and cardiac growth, however, it was not sufficient to trigger pathogenic heart growth. Liu et al’s findings are the first to establish a solid molecular and mechanistic link between miR-222 and cardiovascular benefits suggesting another distinct miRNA as an intriguing therapeutic target, as well as a promising biomarker.

The loss of cardiomyocytes strongly contributes to decreased cardiac function and heart failure, cardiomyocyte proliferation empowers the heart’s regeneration.^44–47^ Understanding the molecular agents, such as miR-222, and pathways that promote cardiomyocyte survival and/or regeneration could have important fundamental and clinical implications.

## Conclusion

Despite the established benefits of exercise, it continues to be underutilized in clinical practice in the fight against cardiovascular diseases. It is hoped that studies highlighting the underlying molecular mechanisms will help in this regard. Furthermore, new therapeutic targets may be identified to decrease residual cardiovascular risk.
